# 

**Published:** 1899-06-03

**Authors:** 


**Notices of Births, Deaths, and Marriages** are inserted in this column at a charge of 2s. 6d. for an announcement not exceeding four lines.

**DEATH.**

The death, on the 26th inst., of Edith Joanna, second daughter of the late John Bonham Carter, of Adhurst St. Mary, Petersfield. Aged 43. (1,871)

### NOTICE TO CORRESPONDENTS.

All MS., Letters, Books for Review, and other matters intended for the Editor should be addressed The Editor, "The Hospital," The Hospital Building, 28 & 29, Southampton Street, Strand, W.C.
The Editor cannot undertake to return rejected MS., even when accompanied by stamped directed envelope.

**Notice to Advertisers and Subscribers.**

All Advertisements, Orders for Copies of The Hospital, and Business Communications should be addressed to **THE MANAGER (Not to the Editor),** The Hospital, The Hospital Building, 28 & 29, Southampton Street, Strand, London, W.C.
The Cost of Subscription, post free, is as follows:—Per week, 2½d.; per quarter, 2s. 8d.; per half-year, 5s. 3d.; per annum, 10s. 6d. Foreign:—Per annum, 15s. 2d., payable, in all cases, in advance.

**NOTICE.—Vol. XXV. of "The Hospital" (October, 1898, to March, 1899), in handsome cloth binding, is now on sale at the Office of this Journal, price 7s. 6d. Cases for binding the Numbers contained in this Volume 1s. 6d. Index to Vol. XXV., 2½d., post free.**
**The Monthly Part for June is now ready. Price 6d., by post 8d.**

### Scraps and Gleanings.

Birmingham Hospital Saturday has been fixed for June 3rd next.

The amount contributed last year to the Oldbury Hospital Saturday Fund was £660.

The ordinary weekly payment at the Lawn Hospital for the Insane, Lincoln, has been raised to 40s.

The report of the Norwood Cottage Hospital for the preceding year acknowledge the receipt of £500 from the Marriott Bequest.

The Archbishop of Canterbury has granted £500 from the Marriott Bequest towards the endowment of the Herne Bay Cottage Hospital.

A large committee has been appointed to investigate and report on the advisability of erecting a new Glasgow parish hospital, to contain 1,200 beds.

The Municipal Committee have passed a resolution asking the Burma Railways Company to contribute to the hospital funds as is done by the Port Trust.

At the ancient hospital of St. Cross, Winchester, the bell of the hospital church is tolled at eight o'clock every Sunday morning, not for service, but in perpetuation of an old custom.

A resolution of condolence to the Duchess of Beaufort in her bereavement has been given expression to by the Bristol Eye Hospital, the Duke of Beaufort having been one of the patrons of the hospitals since 1858.

The estimated cost of erection of the proposed infectious hospital for Hull is £700, according to the plan submitted, and the town clerk is to apply to the Local Government Board for their sanction to a loan of this amount.

During April 151 visits to outside patients were paid by the dispensary sisters of the Royal Hospital for Sick Children, Glasgow. These visits were paid for the purpose of ensuring that the treatment was properly carried out.

Mr. Richard B. Chellew has given 55 guineas towards the fund now being raised for a new Seamen's Hospital for Cardiff.

During the month of February 237 patients were admitted to the Prince Alfred Hospital, Sydney, of whom 126 were discharged as cured.

The "Shilling Fund" initiated by the "Caterham Free Press" in aid of the Caterham Cottage Hospital is progressing but slowly. Perhaps its motto may prove to be "slow and sure."

Over 1,000 patients are treated annually at the Glasgow Samaritan Hospital. The annual cost of maintenance is about £1,800, while the ordinary income amounts to only about £1,600.

The estimated cost of maintaining the City of London Ward in St. Thomas's Hospital is £2,000 per annum. Towards this sum the Mercers' Company have contributed a capital sum of £10,000.

The foundation-stone of the new Homœopathic Hospital at Bromley has been recently laid, and it is hoped that the hospital will be ready for opening in the autumn. The sum now in hand for this object is upwards of £4,000.

Her Grace the Duchess of Marlborough has consented to open the bazaar at Fortescue House, Boys' Home, Twickenham (a branch of the National Refuges for Homeless and Destitute Children), on Friday, June 9th, in aid of the new wing of the home which will be opened on the previous day by their Royal Highnesses the Duke and Duchess of York.

The report of the Friedenheim Hospital (Home of Rest for the Dying), Swiss Cottage, shows that very useful work has been done during the last year. One hundred and five patients were admitted, 24 of whom were discharged, 75 died, and 33 remained in the home. It is hoped that sufficient funds may shortly be forthcoming to enable some better accommodation to be provided for the nurses. At present there is only available for that purpose an amount of £500, and at least four times that amount will be required before the work can be commenced.

28 & 29, SOUTHAMPTON STREET, STRAND, LONDON, W.C.

